# Review of rationale and progress toward targeting cyclin-dependent kinase 2 (CDK2) for male contraception^†^

**DOI:** 10.1093/biolre/ioaa107

**Published:** 2020-06-16

**Authors:** Erik B Faber, Nan Wang, Gunda I Georg

**Affiliations:** 1 Department of Medicinal Chemistry, College of Pharmacy, University of Minnesota–Twin Cities, Minneapolis, MN, USA; 2 Medical-Scientist Training Program, University of Minnesota Medical School, University of Minnesota–Twin Cities, Minneapolis, MN, USA

**Keywords:** contraception, spermatogenesis, kinases, male reproductive tract, meiosis, meiotic arrest

## Abstract

Cyclin-dependent kinase 2 (CDK2) is a member of the larger cell cycle regulating CDK family of kinases, activated by binding partner cyclins as its name suggests. Despite its canonical role in mitosis, *CDK2* knockout mice are viable but sterile, suggesting compensatory mechanisms for loss of CDK2 in mitosis but not meiosis. Here, we review the literature surrounding the role of CDK2 in meiosis, particularly a cyclin-independent role in complex with another activator, Speedy 1 (SPY1). From this evidence, we suggest that CDK2 could be a viable nonhormonal male contraceptive target. Finally, we review the literature of pertinent CDK2 inhibitors from the preclinical to clinical stages, mostly developed to treat various cancers. To date, there is no potent yet selective CDK2 inhibitor that could be repurposed as a contraceptive without appreciable off-target toxicity. To achieve selectivity for CDK2 over closely related kinases, developing compounds that bind outside the conserved adenosine triphosphate-binding site may be necessary.

## Teaser

Cyclin-dependent kinase 2 (CDK2) is a validated and underexploited target for male contraception.

## Introduction

The exploration of nonhormonal targets is considered a new and promising approach to discover and develop highly effective, well-tolerated, and reversible male and female contraceptive agents. Despite this possibility, a nonhormonal male contraceptive agent has not entered clinical trial or progressed toward investigational new drug-enabling preclinical development. The purpose of this review is to explore the evidence and remaining questions surrounding cyclin-dependent kinase 2 (CDK2) as a male nonhormonal contraceptive target. After reviewing the pertinent knockout models and biological evidence, we will briefly review the current literature regarding inhibitor development against CDK2, particularly focusing on selectivity and future approaches.

## Knockout models of *CDK2* are viable but sterile

CDK2 is a member of the CDK family involved in regulating the cell cycle. In mitotic cells, CDK2 is activated by phosphorylation and binds to E-type cyclins to progress from G1 into S phase, then subsequently binds A-type cyclins in S phase to advance the cell cycle [1]. In particular, it was thought that CDK2/cyclin E complexes were necessary for the phosphorylation of retinoblastoma (RB) protein and the release of E2F transcription factors to initiate S phase and DNA synthesis [2, 3]. Despite this role of CDK2 in cell division, unexpectedly two different *CDK2* knockout mouse models have a fully penetrant phenotype of sterility with otherwise normal development and lifespan, but a slightly smaller size after weaning [4, 5]. Anatomical and histological samples, as well as mating behavior, are all normal compared to wild-type mice with the exception of the gonads [4]. Primary mouse embryonic fibroblasts (MEFs) derived from this model are able to proliferate but have a delay into S phase [4, 5]. Further analysis reveals that the CDK2 substrate RB remains phosphorylated, even at sites previously thought to be specific to CDK2, suggesting a compensatory mechanism by another kinase [5]. Phosphorylation of the CDK2 substrate histone H1 in cyclin A immunoprecipitates is additionally observed, suggesting CDK1 compensates for *CDK2* loss in this latter case [5]. From this evidence, CDK2 is necessary for meiosis but not mitosis. It also suggests that selective inhibition of CDK2 could be relatively nontoxic to somatic cells.

The compensatory mechanisms for *CDK2* loss were further explored. Other kinases can compensate for normal CDK2 activity in its absence, particularly CDK1 [6–8]. CDK1 is indispensable and sufficient to drive the cell cycle [6, 7]. In the absence of CDK2, CDK1 binds cyclin E, for example, and recapitulates the natural CDK2 kinase function [8]. Further silencing of *CDK1* in the context of *CDK2* knockout MEFs results in decreased proliferation, suggesting that CDK1 is responsible for compensating *CDK2* loss [8]. Knockout of *CDK2* also results in an earlier transcriptional activation of *CDK1* [9]. Furthermore, full genetic substitution with *CDK2* at the *CDK1* locus results in a nonviable phenotype, emphasizing the unique importance of CDK1 to somatic cell proliferation [9]. However, the meiotic role of CDK2 is dependent on its genetic locus and timing of gene expression, as *CDK2^−/−^* mouse models with the *CDK1^+^*/*CDK2^KI^* haplotype at the *CDK1* locus recapitulates the viable, but sterile phenotype of previous *CDK2^−/−^* mouse models [4, 5, 9]. Overall, a contraceptive agent targeting CDK2 must be selective over CDK1 to have an appropriate toxicological profile.

**Figure 1:**
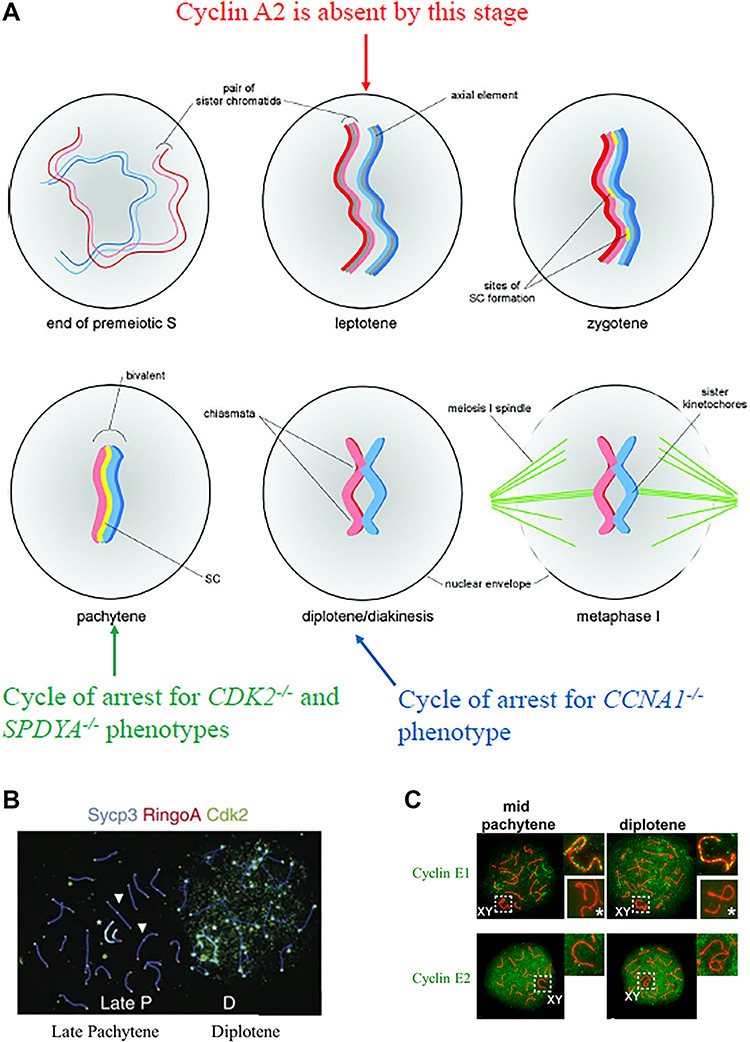
(a) Known CDK2 binding partner cyclin A2 is absent during prophase I. The knockout phenotype of *CDK2* and the knockout phenotype of *SPDYA* both arrest in mid-pachytene. The knockout phenotype of *CCNA1* arrests in diplotene. Image adapted with permission [72]. (b and c) The cellular localization of CDK2 and E-type cyclins do not overlap during pachytene, the subphase of prophase I when *CDK2*^−/−^ cells arrest, where CDK2 is only found on telomeric ends. However, the localization of CDK2 and SPY1 do overlap in pachytene [19, 20].

Male *CDK2^−/−^* mice lack late spermatocytes, spermatids, and sperm [4, 5]. The seminiferous tubules decrease in size but spermatogonia are present, and both Sertoli and Leydig cells appear unaffected [4, 5]. Histological analysis of wild-type and *CDK2^−/−^* testes do not differ until P20, when germ cells complete meiosis I [5]. Notably, germ cells do not differentiate in *CDK2^−/−^* mice [4]. Adult *CDK2^−/−^* mice have atrophic testes 20% the weight and size of their wild-type littermates [5]. In *CDK2^−/−^* cellular models of spermatogenesis, all cells arrest in the mid-pachytene stage of prophase I (Figure 1a) [5]. In particular, unsynapsed chromosomes are observed in this stage, suggesting that *CDK2^−/−^* spermatocytes have a defect in forming the axial element and many chromosomes are unpaired during this stage [5]. While CDK2 is not required for the assembly of the synaptonemal complex, CDK2 regulates homologous pairing and synapsis, as well as formation of the sex body, double-strand break processing, and attachment of telomeres to the nuclear membrane [10, 11].

In female *CDK2^−/−^* mice, severe atrophy of the ovaries is observed even before sexual maturity and increased in severity into adulthood, as *CDK2^−/−^* ovaries are 15–20% the weight and size compared with their wild-type counterparts [4, 5]. The oviduct and uterus are unaffected [5]. No primordial follicles and corpora lutea are observed and oocytes are not developed, suggesting that embryonic development was affected in this tissue, as oocytes normally arrest in prophase I perinatally and continue meiosis later in adulthood [4, 5]. A similar phenotype in wild-type and *CDK2^−/−^* oocytes was observed until the pachytene stage and then diverged greatly by the diplotene stage of prophase I, a substage later than what is observed in spermatogenesis models [5]. In particular, centromeres are randomly distributed around the nuclei in diplotene oocytes whereas they are normally located at discrete locations at this stage [5].

In adult men, spermatogenesis and meiosis are ongoing processes, unlike oogenesis, which begins in utero and arrests in meiosis I. Because CDK2 acts in an early stage of meiosis I, it is a validated contraceptive target for men but remains unclear in women, as a reversible therapeutic targeting CDK2 might be inappropriately timed to affect oogenesis in adult women. To answer this question, two oocyte-specific conditional knockout mouse models were made: one for *CDK2* (Oo*CDK2^−/−^*) and the other for *CDK1* (Oo*CDK1^−/−^*) [12]. In particular, primordial and further developed follicles are devoid of their respective proteins in these two models, and ovaries without *CDK1* or *CDK2* are morphologically indistinguishable from their wild-type counterparts. Ovulation and formation of corpora lutea are also unaffected. However, only Oo*CDK1^−/−^* mice are infertile whereas Oo*CDK2^−/−^* mice normally develop oocytes and are as fertile as their wild-type counterparts with normal litter sizes [12]. CDK1 is necessary for germinal vesicle breakdown, as triggered by luteinizing hormone to resume meiosis, whereas CDK2 is not. Loss of *CDK2* does also not affect the sequential arrest in meiosis II before fertilization [12]. Rescue of the Oo*CDK1^−/−^* genotype with *CDK1* mRNA injection resumes meiosis appropriately, further signifying that CDK1 is responsible for resumption of meiosis postnatally [12]. This would suggest that a contraceptive targeting CDK2 could be effective in adult men, but unlikely for adult women.

## CDK2 is necessary in spermatogenesis via a cyclin-independent mechanism

While the precise role of CDK2 in meiosis is unknown, it appears that its activation is cyclin-independent, unlike its role in interphase. Because CDK2 is known to bind to cyclins A and E in particular, and a role for cyclins in meiosis is established, it was assumed the CDK2 bound to cyclin was necessary to advance meiosis.

In both mice and humans, CDK2 binds two isoforms of cyclin A—cyclin A1 (CCNA1) and cyclin A2 (CCNA2). The cyclin A1 isoform is primarily expressed in the germ line and restricted to meiotic cells. It is distributed in the nucleus after mid-pachytene and then localized on the telomeres in late diplotene, persisting through metaphase of the first meiotic division but not though the second. Cyclin A1 demonstrates haploinsufficiency for mouse fertility and has been hypothesized to be responsible for instances of human oligospermia [13]. Antibodies against cyclin A1 precipitate both CDK1 and CDK2 in testicular lysates [14]. However, knockout models of *CCNA1* arrest in the later diplotene stage with the disappearance of the synaptonemal complex, differing in phenotype from that of *CDK2* knockouts, which arrest in the pachytene stage where the synaptonemal complex is still intact (Figure 1a) [15]. Within the testis of *CCNA1^−/−^* models, CDK1 activity decreases by 80% while CDK2 activity only decreases modestly, suggesting that cyclin A1 acts primarily through CDK1 in the testis [15]. Immuno-depletion of cyclin A1 in testis extracts also does not significantly alter the overall activity of CDK1 or CDK2, suggesting neither kinase is strongly dependent on cyclin A1 [15]. Finally, cyclin A1 and CDK2 do not colocalize in prophase I of spermatogenesis, indicating that cyclin A1 is not the pertinent binding partner of CDK2 [16].

In contrast, cyclin A2 is ubiquitously expressed in dividing somatic cells and restricted to the premeiotic S-phase in germ cells. Knockout models of *CCNA2* are embryonically lethal, making its precise role in spermatogenesis difficult to study [17]. However, it is present only in spermatogonia and preleptotene spermatocytes (Figure 1a) [13]. In wild-type testicular lysates, anticyclin A2 antibodies precipitates CDK2 but not CDK1, and anti-CDK2 antibodies precipitate cyclin A2 as well as other polypeptides [13]. The presence of CDK1 and its ability to bind cyclin A2 may compensate for the loss of CDK2 in the pre-pachytene stages of *CDK2^−/−^* models. Since cyclin A2 is absent for all of prophase I and therefore is absent during the time *CDK2^−/−^* spermatocytes arrest in mid-pachytene, it is unlikely CDK2-cyclin A2 is the pertinent complex responsible for *CDK2^−/−^* spermatocyte arrest.

CDK2 also binds E-type cyclins, of which there are two in mammals, cyclins E1 and E2. Cyclins E1 and E2 are thought to have redundant functions in meiosis, as knockout mouse models of either cyclin E gene individually result in viable offspring. However, male *CCNE2^−/−^* mice have reduced fertility, with a decreased testis size and a sperm count 50% to their wild-type counterparts [18]. In contrast, double knockout models of *CCNE1* and *CCNE2* are embryonically lethal and conditional double knockout models yield a sterile and azoospermic phenotype in male mice [18, 19]. From this, the role of E-type cyclins in spermatogenesis was explored. E-type cyclins help spermatocytes progress through prophase I [19]. Cyclin E1 appears at pachytene of prophase I until diplotene while cyclin E2 is present from preleptotene and increases throughout most of prophase I [19]. Cyclin E1 is located on the sex chromosomes while cyclin E2 is not [19]. Although loss of *CCNE1* does not lead to infertility, it does disrupt the formation and progression of synapsis [19]. In contrast, loss of *CCNE2* does not affect fertility until the diplotene stage, but does result in heterologous chromosomal associations during pachytene involving a “one-to-one” chromosome connection on the telomeric ends [19]. E-type cyclins are also necessary for double-strand break repair as well as telomere structural integrity and stability [19].

While E-type cyclins and CDK2 appear to be acting early in prophase I, it is unlikely their function is co-dependent. For example, CDK2 is distinctly localized on the telomeres and recombination nodules throughout prophase I [10]. This is demonstrated in Figure 1b, where the chromosomes are highlighted by red SYCP3 and CDK2 is highlighted in green at the ends of these chromosomes during the stages of prophase I [20]. In contrast, Figure 1c shows green E-type cyclins diffusely spread throughout the nucleus, suggesting that E-type cyclins and CDK2 are not colocalized during prophase I [19]. However, co-immunoprecipitation studies of cyclins E1 and E2 reveal an interaction with CDK2 [19]. While a catalytic function was not measured from these precipitates, it is suggested that E-type cyclins help CDK2 localize to the telomeres. *CCNE1^−/−^* spermatocytes had similar CDK2 telomeric localization as wild-type spermatocytes, *CCNE2^−/−^* spermatocytes had a 50% reduction in CDK2 telomeric localization, and double knockout *CCNE1^−/−^ CCNE2^−/−^* spermatocytes had a 93% reduction in CDK2 telomeric localization. While this would suggest that E-type cyclins are important for the appropriate localization of CDK2, it is unclear whether E-type cyclins localize CDK2 in particular or serve to stabilize the telomere in general. Whether E-type cyclins perform catalysis in association with CDK2 or serve a scaffolding function for CDK2 in spermatogenesis also remains to be determined. Finally, the phenotypes of E-type cyclin loss and *CDK2^−/−^* differ. Complete ablation of E-type cyclin function in spermatocytes arrests cells in the early pachytene, near but distinct from the mid-pachytene arresting phenotype of *CDK2^−/−^* spermatocytes [5, 19]. Only 1.5% of E-type cyclin depleted spermatocytes progress into mid-pachytene [19].

## CDK2 is necessary in meiosis via a complex with SPY1

**Figure 2:**
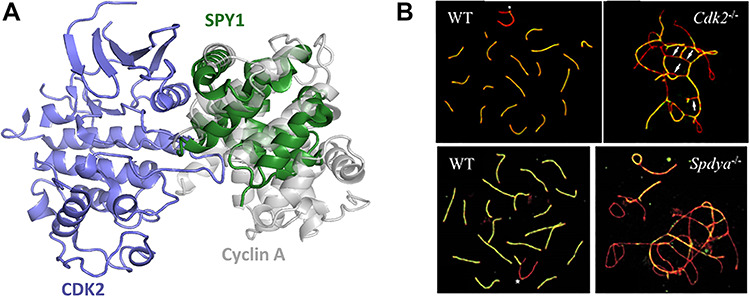
(a) SPY1 interacts with CDK2 along the same interface as cyclin (overlay of PDB IDs 1FIN and 5UQ1). (b) The chromosomal phenotypes of *CDK2*^−/−^ and *SPDYA*^−/−^ show nearly identical arresting phenotypes in mid-pachytene [20].

Recently, an additional role of CDK2 in meiosis has been established. The protein Speedy 1 (SPY1), also known as Ringo A, can activate CDK2 by binding to the same site as cyclins (Figure 2a) [21]. Unlike cyclin activation, SPY1 activation of CDK2 does not require phosphorylation to initiate its catalytic function [22]. SPY1 is expressed in all tissues, but its expression is only substantially increased in the testis (20 ± fold higher) [23]. *SPDYA^−/−^* mice have the same viable but sterile phenotype as of *CDK2^−/−^* mice, with no additional observed abnormalities [20]. The hypoplastic testes from *SPDYA^−/−^* mice are four times smaller than their wild-type counterparts and while spermatogonia exist in the mutant mice, neither spermatozoa nor spermatids are observed [20].

In particular, *SPDYA^−/−^* spermatocytes and oocytes arrest in the pachytene stage of prophase I, as is observed with *CDK2^−/−^* spermatocytes (Figure 2b) [20]. *SPDYA^−/−^* spermatocytes have more double-strand breaks and no crossing-over events, suggesting that SPY1 affects late recombination and homologous chromosome pairing [20]. Additionally, *SPDYA^−/−^* spermatocytes show nonhomologous chromosome pairing, failure of the telomeres to attach to the nuclear membrane, and telomere fusion, just like *CDK2^−/−^* spermatocytes [5, 10, 20]. SPY1 and CDK2 colocalize at the telomeres in spermatocytes from leptotene throughout pachytene, even on the asynapsed sex chromosomes [20]. Interestingly, CDK2 does not localize on the telomeres in *SPDYA^−/−^* spermatocytes, suggesting that SPY1 may help to localize CDK2 to the telomere. In wild-type spermatocytes, antibodies against SPY1 co-immunoprecipitated CDK2 and vice versa. In *SPDYA^−/−^* spermatocytes, the activity of immunoprecipitated CDK2 decreased by 70%, highlighting the importance of the CDK2-SPY1 catalytic complex [20]. Loss of localization and decreased CDK2 activity are thought to be the causes of infertility in *SPDYA^−/−^* mice.

While the substrate profile of CDK2-SPY1 is incomplete, CDK2-SPY1 can phosphorylate the protein SUN1 in vitro [11, 20]. Furthermore, *SUN1* knockout mouse models are very similar to the *SPDYA* knockout and *CDK2* knockout models, demonstrating nonhomologous pairing, increased double-strand breaks, and loss of telomeric attachment to the nuclear membrane [5, 10, 20, 24]. SUN1 is important for localizing telomeres to the nuclear membrane in prophase I [24–26]. In *SPDYA^−/−^* spermatocytes, SUN1 does not localize to telomeres as it normally does [20]. In wild-type spermatocytes, SUN1 associates with TERB1, another protein important for telomeric localization to the nuclear membrane [27]. TERB1 localization is unperturbed on telomeres in *SPDYA^−/−^* spermatocytes [20]. It is hypothesized that phosphorylation of SUN1 by CDK2-SPY1 is necessary for the association of SUN1 with TERB1. This could be one mechanism by which the CDK2-SPY1 complex is necessary in spermatogenesis. The high concentration of SPY1 in the testis, coupled with a relatively healthy *SPDYA* knockout model and validated mechanism in spermatogenesis via CDK2 binding, suggests that targeting a SPY1-CDK2 complex could be a viable strategy to develop a safe, nonhormonal male contraceptive.

## CDK2 inhibitors

During the last two decades, CDK2 inhibitors have not been developed for contraception but instead predominantly to treat cancer. While 18 CDK2 inhibitors have entered clinical trials, none have been approved by the Food and Drug Administration. In the final sections of this review, we will summarize the information about CDK2 inhibitors that have entered clinical trials, selective CDK2 inhibitors in preclinical development, allosteric CDK2 inhibitors, and selected other CDK2 inhibitors with a view toward their potential use as contraceptive agents.

## CDK2 inhibitors in the clinic

Eighteen nonselective CDK inhibitors (Table 1; Supplementary data, Figure S1) have entered clinical trials for the treatment of different types of cancer; 10 are currently in clinical trials and the others were terminated after phase I or II trials due to undesirable off-target toxicity and poor pharmacokinetic properties. First-generation CDK inhibitors include alvocidib, a flavonoid alkaloid, BMS-387032, a compound with a 2-aminothiazole-5-thiol core, PHA-793887, featuring a 1*H*-pyrazol-3-amine scaffold, and (*R*)-roscovitine, a purine. These inhibitors failed to show any significant clinical advantages, which has been attributed to off-target effects and inappropriate tumor type selection for testing [28]. While (*R*)-roscovitine also inhibits CDK1/5/7/9, specific inhibition of CDK2 by this compound was directly linked to cancer cell apoptosis [29]. A phase II study of (*R*)-roscovitine for Cushing disease was initiated in 2018 and is currently (April 2020) recruiting patients. The five other recorded phase I/II studies of (*R*)-roscovitine were either withdrawn or terminated at an early stage for unknown reasons [30]. Similarly, the CDK1/2/4/5/7/9-targeting compound alvocidib was investigated in phase I/II studies, but did not show significant clinical advantages [31]. However, phase I/II studies of combination therapies of alvocidib and venetoclax were initiated for the treatment of acute myeloid leukemia [32]. BMS-387032 is a CDK2/7/9 inhibitor that underwent two phase I trials for the treatment of advanced solid tumors and B-cell malignancies [33]. PHA-793887 was investigated in a phase I clinical trial in 2008 to study dose-escalation in patients with acute myeloid leukemia and myelodysplastic syndromes [34]. However, this study was terminated early for unknown reasons. In order to achieve a higher selectivity for CDK1 and CDK2 and to further increase potency, a second-generation of CDK inhibitors were developed. In particular, dinaciclib targets CDK1/2/5/9 and has been most studied in the clinic among all second-generation CDK inhibitors, tested as a single agent or in combination with other therapies [35]. Dinaciclib exhibited activity against a broad range of tumor cell lines including various breast, prostate, colon, and lung cancer cell lines [36]. Dinaciclib is currently in phase I trials for the treatment of multiple myeloma, diffuse large B-cell lymphoma, and triple negative breast cancer. Various phase II and III trials of dinaciclib have been completed for the treatment of nonsmall cell lung cancer, advanced breast cancer, acute myeloid leukemia, acute lymphocytic leukemia, stage IV melanoma, and mantle cell lymphoma [37–39]. The only completed phase III study of dinaciclib is a combination therapy of dinaciclib and ofatumumab for the treatment of refractory chronic lymphocytic leukemia in 2014, which concluded that dinaciclib had an acceptable safety and tolerability profile in these patients [40]. The most common adverse effects of dinaciclib were neutropenia (35%), thrombocytopenia (20%), decreased neutrophil count (20%), febrile neutropenia (10%), pneumonia (5%), and sepsis (5%) [40]. While this side effect profile may be acceptable for patients with cancer, it is unlikely to be adequate for an approved contraceptive.

**Table 1:** CDK2 inhibitors that have entered clinical trials.

CDK2 inhibitors in the clinic	Highest Phase	Conditions	Targets	References
Alvocidib hydrochloride (flavopiridol hydrochloride, DSP-2033, HL-275, HMR-1275, L86-8275, MDL-107826A, or NSC-649890)	Phase II^a^	Leukemia, acute myeloid and myelodysplasia	CDK1/2/4/5/7/9, BCL-2, MCL-1 and XIAP	[73]
AT7519	Phase II^a^	Refractory solid tumors and lymphoma	CDK1/2/5/9 and GSK3$\beta$	[74]
BMS-387032 (SNS-032)	Phase II	Multiple myeloma, hematological cancer, and solid tumors	CDK2/7/9	[75]
Couroupitine B (NSC-105327, indigo red, indigopurpurin, or indirubin)	Phase I	Myeloid leukemia	CDK1/2/4/5 and GSK3$\beta$	[76]
Dinaciclib (MK-7965, NSC-727135, or SCH-727965)	Phase III^a^	Advanced breast cancer, nonsmall cell lung cancer, acute lymphocytic leukemia, acute myeloid leukemia, mantle cell lymphoma, and stage IV melanoma	CDK1/2/5/9	[36]
Fadraciclib (CYC-065)	Phase I^a^	Advanced solid tumors, chronic lymphocytic leukemia, acute myeloid leukemia, and myelodysplastic syndromes	CDK2/5/9	[77]
FN-1501	Phase I^a^	Acute myeloid leukemia and solid tumor	CDK2/4/6 and FLT3	[78]
7-Hydroxystaurosporine (KRX-0601, KW-2401, NSC-638850 or UCN-01)	Phase II	Small cell lung cancer, leukemia, lymphoma, leukemia, melanoma, ovarian cancers, and non-Hodgkin’s lymphoma	CDK1/2/4/6, CHK1/2, PI3K, NHE, PDK1, and PKC	[79]
Milciclib (PHA-848125 or TZLS-201)	Phase II^a^	Hepatocellular carcinoma and thymus	CDK1/2/4/5/7, WEE1/2, and TRKA	[48, 80]
PF-06873600	Phase II^a^	Breast cancer and ovarian cancer	CDK2/4/6	[81]
PHA-690509	Phase I	Cancer	CDK2	[82]
PHA-793887	Phase I	Leukemia and solid tumors	CDK1/2/4/5	[83]
Roniciclib (Bay-1000394)	Phase II	Small cell lung cancer, ovarian cancer, and solid tumors	CDK1/2/3/4/9, FLT4, AURK1, JAK2/3 and MAP3K9	[47, 84]
(*R*)-Roscovitine (Seliciclib, CYC-202 or NSC-701554)	Phase II^a^	Cushing syndrome, fibrosis, rheumatoid arthritis, and solid tumors	CDK1/2/5/7/9	[29]
R-547	Phase I	Solid tumors	CDK1/2/4	[85]
TP-1287	Phase I^a^	Myeloid leukemia and solid tumors	CDK1/2/4/6/7/9, BIRC5, Mcl-1, Bcl-2, and XIAP	[86]
Voruciclib hydrochloride(P-1446)	Phase I^a^	Diverse advanced solid tumors, hematologic malignancies and relapsed/refractory B-cell malignancies	CDK1/2/4/9	[87]
ZK-304709 (ZK-CDK)	Phase I	Solid tumors	CDK1/2/4/7/9 and FLK1/4	[88]

^a^Under active development.

7-Hydroxystaurosporine, an analog of the natural product staurosporine, is a CDK1/2/4/6 inhibitor that completed phase I studies for the treatment of relapsed or refractory acute myeloid leukemia and chronic myelogenous leukemia, which demonstrated that it can be safely administered but lacks clinical efficacy [41, 42]. Moreover, a phase II study of 7-hydroxystaurosporine for the treatment of relapsed T-cell lymphomas was terminated [43]. AT-7519 is a potent CDK1/2/5/9 inhibitor that was discontinued in phase II trials for the treatment of advanced solid tumors and multiple myeloma due to the low objective response rates for reduction in tumor burden [44–46]. Roniciclib is a pan-CDK inhibitor that underwent phase II clinical trials for the treatment of small cell lung cancer [47]. However, roniciclib demonstrated an unfavorable risk–benefit profile in patients with small cell lung cancer, leading to premature termination of the study [47]. Another CDK inhibitor, milciclib, is currently undergoing phase II clinical trials for the treatment of thymic and hepatocellular carcinomas [48, 49]. Finally, the dual CDK2/4/6 and FT3 inhibitor FN-1501 is in a phase I trial for the treatment of acute myeloid leukemia and solid tumors [50].

Information about other drugs, which have not completed any clinical trials, are summarized in Table 1. Of all the CDK2 inhibitors that have entered clinical trials, none are selective or safe enough to be repurposed for contraceptive indications.

## Selective type I inhibitors

The discovery of CDK2 inhibitors has primarily focused on targeting the adenosine triphosphate (ATP)-binding site (type I inhibitors). In Table 2, inhibitors are summarized that are in preclinical development and have appreciable selectivity for CDK2 over other CDKs. Their chemical structures and code names are shown in Supplementary data, Figure S2. The inhibitors compound 73, CCT068127, NU2058, NU6102, and purvalanol B are purine analogs mimicking the adenosine in ATP. Several are strong inhibitors, such as NU6102 with an IC_50_ of 6.0 nM [51]. The introduction of a 4-sulfamoylalanilino group to the C2 position of NU2058 provided analog NU6102 that has enhanced selectivity for CDK2 to 50-fold greater over CDK1/4/5, which all share close structural homology with CDK2. Because CDK1 is necessary for somatic cells, a contraceptive CDK2 inhibitor should not inhibit CDK1. Furthermore, some kinases such as CDK7/CDK9 phosphorylate RNA polymerase II and therefore the inhibition of these off-target kinases may conceal the true pharmacological effects of purported CDK2 inhibitors including dinaciclib, (*R*)-roscovitine, and BMS-387032 [52]. Purine-derived compound 73, carrying a biphenyl moiety at the 6-position, has superior selectivity for CDK2 over CDK1 (2000-fold) compared with (*R*)-roscovitine and dinaciclib, but still inhibits CDK9 [53]. Purvalanol A and purvalanol B are purines with better selectivity toward CDK2 than (*R*)-roscovitine and alvocidib, which was achieved by modification of the 2-, 6-, and 9-positions of the purine core [54].

**Table 2:** Inhibition by selective ATP-site CDK2 inhibitors against CDKs.

Compounds	IC_50_ (nM)								References
	CDK2/cyc A	CDK1/cyc A	CDK3/cyc E	CDK4/cyc D1	CDK5/p25	CDK6/cyc D1	CDK7/cyc H	CDK9/cyc K	
AZD5438	6	16	-	-	-	-	-	20	[58]
CCT068127	110(10^a^)	1100^b^	-	4800	70^d^	6200^e^	520	90	[55]
Compound 51	1.1	7.6	38	4.0	1.5	6.6	>1000	13	[56]
Compound 73	44	86 000^b^	-	26^c^	-	-	28^c^	25	[53]
NU2058	17	26	-	-	-	-	-	-	[89]
NU6102	6^a^	146^b^	-	184	122	-	2530	4120	[51]
Purvalanol B (NG-60)	6(9^a^)	6^b^	-	10 000	6^d^	-	-	-	[54]
SCH-546909	14	-	-	1420	-	-	-	-	[59]
SU9516	22	40	-	200	-	-	-	-	[57, 90]

- Not available.

^a^IC_50_ against CDK2/cyclin E.

^b^IC_50_ against CDK1/cyclin B.

^c^% inhibition at 100 $\mu$M.

^d^IC_50_ against CDK5/p35.

^e^IC_50_ against CDK6/D3.

More selective CDK2 inhibitors with other scaffolds have also been developed. CCT068127 has improved selectivity toward CDK2 compared with the parent (*R*)-roscovitine, but it inhibits CDK5/9 [55]. CCT068127 forms additional hydrogen bonding interactions with the DFG-motif, explaining its improved potency compared with (*R*)-roscovitine. Thiazole core compound 51 displays high selectivity for CDK2 and CDK5 over other CDKs [56]. SU9516 has a tri-substituted indolinone core that is 2-fold selective for CDK2 over CDK1 and more than 20-fold selective for CDK2 over CDK4 [57]. AZD5438 contains an imidazole core and is selective for CDK1/2/9 [58]. SCH-546909 is derived from a natural product and exhibits 10-fold selectivity for CDK2 over CDK4, but less than 2-fold selectivity for CDK2 over CDK1 [59]. In summary, because no CDK2 inhibitor with substantial selectivity over other CDKs exists to date, if these compounds were to be used chronically, for example, as a contraceptive agent, then the off-target effects present a significant problem.

## Type II CDK2 inhibitors

Inhibition of CDK2 can also be achieved through stabilization of an inactive conformation with the DFG motif facing out (DFG-out) toward the solvent (type II). The energy differences between the conformations of the DFG-out activation loops of kinases [60, 61] offer an opportunity to obtain type II inhibitors with improved selectivity compared with type I inhibitors. K03861, an aminopyrimidine-phenyl urea inhibitor (Supplementary data, Figure S3), was identified as a CDK2 type II inhibitor with a K_d_ of 53 nM [62] and is the first type II inhibitor of CDK2. K03861 competes with cyclin binding and stabilizes the inactive state of CDK2 by binding to the ATP-binding site and reaching toward a hydrophobic pocket located between the C-lobe and the alpha-C-helix (Supplementary data, Figure S4, PDB ID: 5A14). However, this compound was promiscuous and therefore not selective for CDK2.

## Type III and IV allosteric inhibitors

Although a majority of the previous CDK2-targeting efforts focused on the ATP-binding site, achieving high selectivity for CDK2 against other kinases in this manner is difficult due to the highly conserved nature of the kinase ATP-binding site, especially within the CDK family. The discovery of allosteric pockets provides an opportunity to attain a higher degree of selectivity toward CDK2, as these pockets are often less conserved.

**Figure 3:**
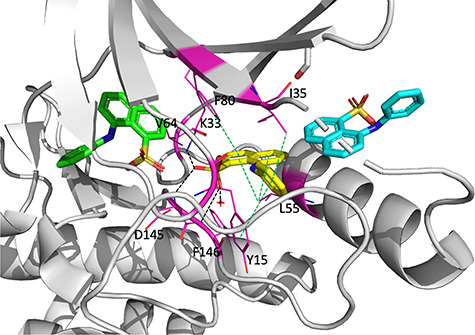
The co-crystal structure of three molecules of ANS bind to CDK2 (PDB ID: 3PXQ) where one of them binds to the ATP-binding site (green), and the other two bind deeper within the allosteric pocket (yellow and cyan). The red cross is a water molecule. Black dotted lines indicate hydrogen bonds and salt bridges. Green dotted lines indicate hydrophobic interactions. Magentas residues interact with the ANS molecule deep within the allosteric pocket (yellow).

Four allosteric pockets in CDK2 have been identified crystallographically (Table 3) with ligands bound in each of them [63–65]. Additionally, two allosteric pockets were putatively discovered by computational methods [66, 67]. The best characterized allosteric pocket binds 8-anilino-1-naphthalene sulfonate (ANS), as confirmed by X-ray crystallography [63]. In the allosteric pocket, an ANS molecule binds adjacent to the ATP-binding site and the C-helix (Figure 3, PDB ID: 3PXQ). The sulfonic acid moiety of ANS forms hydrogen bonds and salt bridges through interactions with K33, D145, and F146, whereas the naphthalene and aniline rings form hydrophobic interactions with Y15, I35, L55, V64, and F80 (Figure 3). A second ANS molecule binds adjacent to the first in the allosteric pocket and also interacts with C-helix residues (Figure 3). The major residues interacting with the second ANS molecule are I52, L76, K56, and H71 (Figure 3). At high concentrations of ANS, a third ANS molecule binds in the ATP-binding site (Figure 3).

**Table 3:** Allosteric inhibitors of CDK2.

Compounds	PDB ID	The binding pocket of CDK2	K_d_ ($\mu$M)	CDK2 IC_50_ ($\mu$M)	IC_50_ ($\mu$M) of cell viability assays	References
ANS	3PXZ, 3PY1, 3PXF, 3PXQ, 4EZ7	ANS pocket	37	91	-	[63]
B2	-	Noncatalytic pocket near the interface of the CDK2/cyclin A3	-	52	85^a^	[66]
Compound 1	5OSJ	An allosteric pocket adjacent to the cyclin binding interface	-	-	-	[65]
Compound 2	5FP6	The second ANS pocket	-	-	-	[64]
Compound 3f	-	ANS pocket	43	10	-	[68]
DAALT	-	Interface of CDK2/cyclin E	0.5	-	-	[67]
DPIT	-	ANS pocket	-	-	-	[70]
FLI-06	-	ANS pocket	-	71	4^b^	[69]

- Not available.

^a^IC_50_ with A549.

^b^IC_50_ with MDA-MB231.

The experimental K_d_ of ANS for CDK2 is 37 μM and its inhibitory potential against the active CDK2-cyclin A complex is weak with an IC_50_ = 91 μM. Notably, the ANS pocket is inaccessible when CDK2 binds to cyclin A/E. Therefore, inhibitors targeting this binding site require high affinity to outcompete cyclins and drive the equilibrium back to inactive free CDK2. Since discovery of this previously unrecognized allosteric pocket, several inhibitors with different scaffolds targeting this pocket have been reported, including FLI-06, compound 3f, and DPIT (Supplementary data, Figure S5), although none of which have been confirmed by structural methods to bind in this pocket. Compound 3f has an IC_50_ = 10 μM, representing the best inhibition against CDK2 of this series to date [68]. In addition, compound 3f inhibits wild-type epidermal growth factor receptor (EGFR) as well as carcinogenic mutant L858R/T790M EGFR, with IC_50_ values of 50 and 10 μM, respectively. Similarly, increasing concentrations of FLI-06 displaces ANS, suggesting that it binds in the same pocket [69]. In cancer cell lines, FLI-06 inhibits cell viability with an IC_50_ = 4 μM against MDA-MB231 cells and IC_50_ = 4.5 μM against ZR-75-1 cells. The group that discovered FLI-06 also developed compound 3f. DPIT, a previously reported anti-HSV-1 agent, was later suggested to bind the ANS pocket via docking studies [70]. None of these published allosteric inhibitors, however, has been crystallographically confirmed to bind to the ANS allosteric pocket. The only crystallographically confirmed fragment that binds to the ANS pocket is compound 2 [64]. The alignment in Supplementary data, Figure S6 shows that compound 2 binds in a similar location as the ANS molecule in the second allosteric ANS site within the allosteric pocket.

The other allosteric pockets were discovered computationally at the interface of CDK2 and cyclin E, next to the T-loop [67, 71]. Short 5-mer peptide DAALT bind to the interface allosteric pocket with the strongest affinity of K_d_ = 0.5 μM to prevent the protein–protein interaction of CDK2 and cyclins [67]. Docking studies indicated that C177, K178, and Y180 are three key residues with the highest contribution toward binding these small peptides. These peptide inhibitors were suggested to induce the similar conformational change as cyclins, although this was never confirmed structurally [67]. Another allosteric pocket near the CDK2/cyclin A3 interface has been suggested [66]. Compound B2 (Supplementary data, Figure S5), obtained from a virtual high throughput screen, inhibits CDK2/cyclin A3 interaction with an IC_50_ = 52 μM and exhibits weak antiproliferative activities against A549, HepG2, and MDA-MB-231cell lines [66]. However, compound B2 contains a PAINS scaffold and an imine moiety that may be unstable in aqueous solution. Considering these factors and the lack of structural data for the presence of the pocket where compound B2 binds, more data are needed to confirm these results.

A crystallographically verified (PDB ID: 5OSJ) type IV acryl amide covalent inhibitor, (compound 1; Supplementary data, Figure S5) binds to an allosteric pocket adjacent to the cyclin binding interface, forming a covalent bond with Cys177, a conserved residue in CDK2 not present in the other CDK family members [65]. However, the lead covalent inhibitor exhibits weak inhibition against CDK2-cyclin A2 with 83% inhibition at 0.5 mM. Covalent inhibitors may be acceptable as contraceptive agents if they are selective, but further studies are needed to validate this assertion.

## Remaining questions about CDK2 as a contraceptive target

While the importance of CDK2 in spermatogenesis is clear, its appeal as a contraceptive target remains more uncertain. The *CDK2^−/−^* mouse model suggests that a CDK2-selective inhibitor would have minimal off-target effects due to compensation in somatic cells by CDK1. However, it is unclear what effect prolonged CDK2 inhibition would have on a healthy adult, as CDK2 aids in repairing DNA double-strand breaks, known causes of numerous cancers. Because spermatocytes that arrest in mid-pachytene undergo apoptosis, the reversibility of a selective CDK2 inhibitor as a contraceptive agent needs to be confirmed. However, because spermatogonia are unaffected, a selective CDK2 inhibitor should be reversible. Additionally, a CDK2 inhibitor that interacts with protein binding partners (e.g., cyclins) could serve to not only inhibit CDK2 but also sequester cyclins, which could be toxic, as other CDK family members like CDK1 might not be activated. An ideal CDK2 inhibitor for contraception could therefore be a PROTAC molecule, which degrades CDK2 and recapitulates the *CDK2^−/−^* mouse model, or an inhibitor that disrupts the ability of CDK2 to bind to partner proteins like the cyclins or SPY1. Such an inhibitor could bind the ATP site or an allosteric site, as long as it is negatively cooperative with partner protein binding. Another option is to develop an inhibitor that targets the CDK2-SPY1 complex while not affecting the complexes of phosphorylated CDK proteins with cyclins.

Finally, the drug administration schedule and acceptability need to be considered. Because the role of CDK2 in spermatogenesis is at the beginning of meiosis I and the entire spermatogenesis process takes over a month in adult males, a selective CDK2-targeting contraceptive would not have an immediate contraceptive effect. The pharmacokinetic properties of a selective CDK2 inhibitor must be considered when dosing to ensure a high level of efficacy as a contraceptive. Since this would be a medication that will to be taken by otherwise healthy individuals chronically, the tolerance for undesirable side effects will be very limited.

## Supplementary Material

fig_S1_ioaa107

fig_S2_ioaa107

fig_S3_ioaa107

fig_S4_ioaa107

fig_S5_ioaa107

fig_S6_ioaa107
