# Supplementary material for: Review of rationale and progress toward targeting cyclin-dependent kinase 2 (CDK2) for male contraception
Source: Biol Reprod. 2020 Jun 16;103(2):357–67. doi: 10.1093/biolre/ioaa107 (PMC7523694; doi:10.1093/biolre/ioaa107)
Supplement: fig_S1_ioaa107 [file fig_s1_ioaa107.pdf]

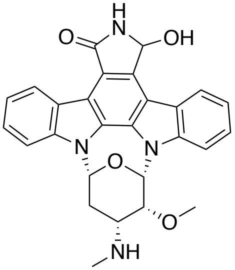

7-Hydroxystaurosporine

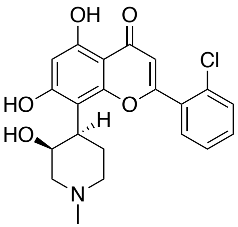

Alvocidib

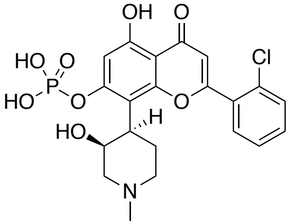

TP-1287

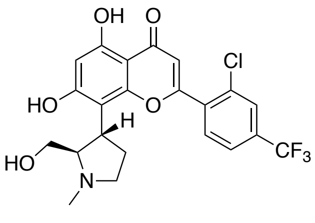

Voruciclib

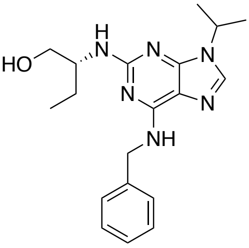

(*R*)-Roscovitine

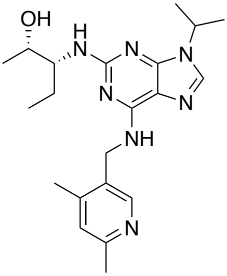

Fadraciclib

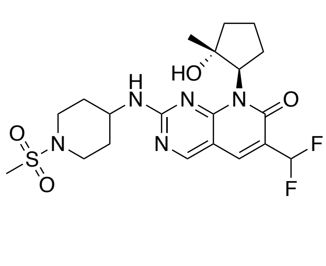

PF-06873600

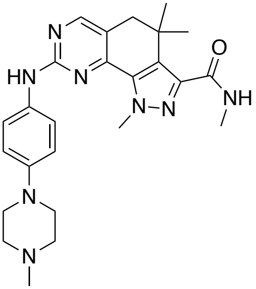

Milciclib

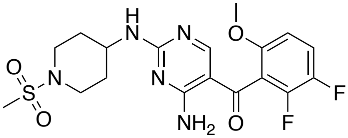

R-547

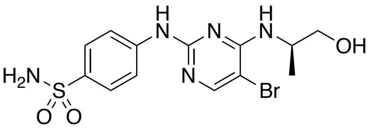

ZK-304709

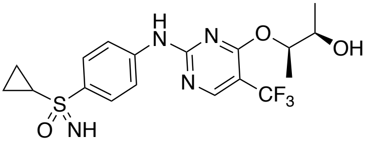

Roniciclib

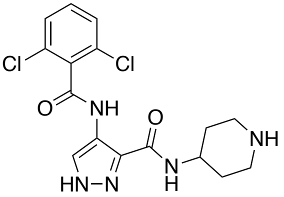

AT7519

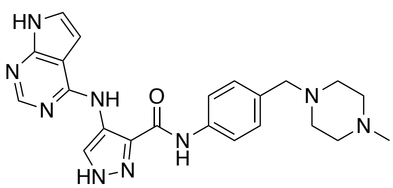

FN-1501

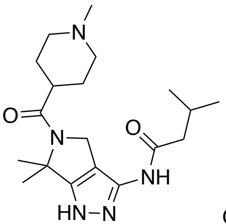

PHA-793887

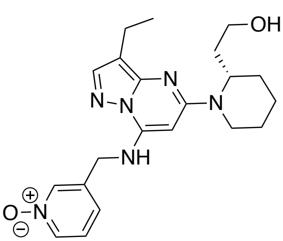

Dinaciclib

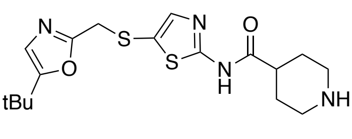

BMS-387032

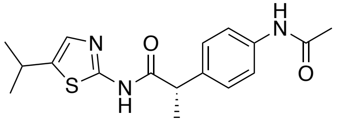

PHA-690509

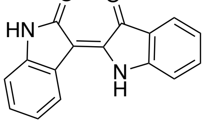

Couroupitine B
