# Supplementary figures and images for: Review of rationale and progress toward targeting cyclin-dependent kinase 2 (CDK2) for male contraception
Source: Biol Reprod. 2020 Jun 16;103(2):357–67. doi: 10.1093/biolre/ioaa107 (PMC7523694; doi:10.1093/biolre/ioaa107)

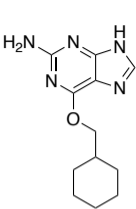

**NU2058**

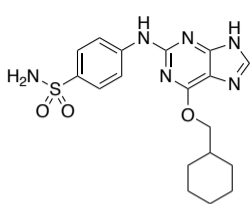

**NU6102**

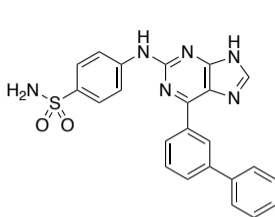

**Compound 73**

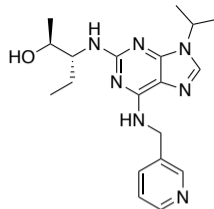

**CCT068127**

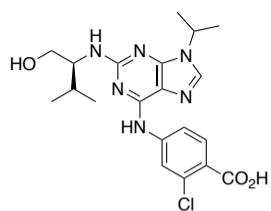

**Purvalanol B**

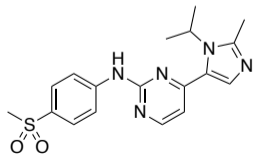

**AZD5438**

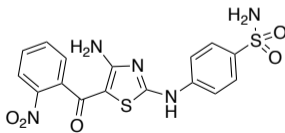

**Compound 51**

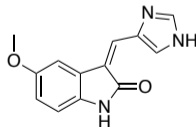

**SU9516**

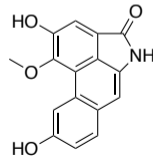

**SCH-546909**

Supplement: fig_S2_ioaa107 [file fig_s2_ioaa107.pdf]

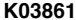

Supplement: fig_S3_ioaa107 [file fig_s3_ioaa107.pdf]

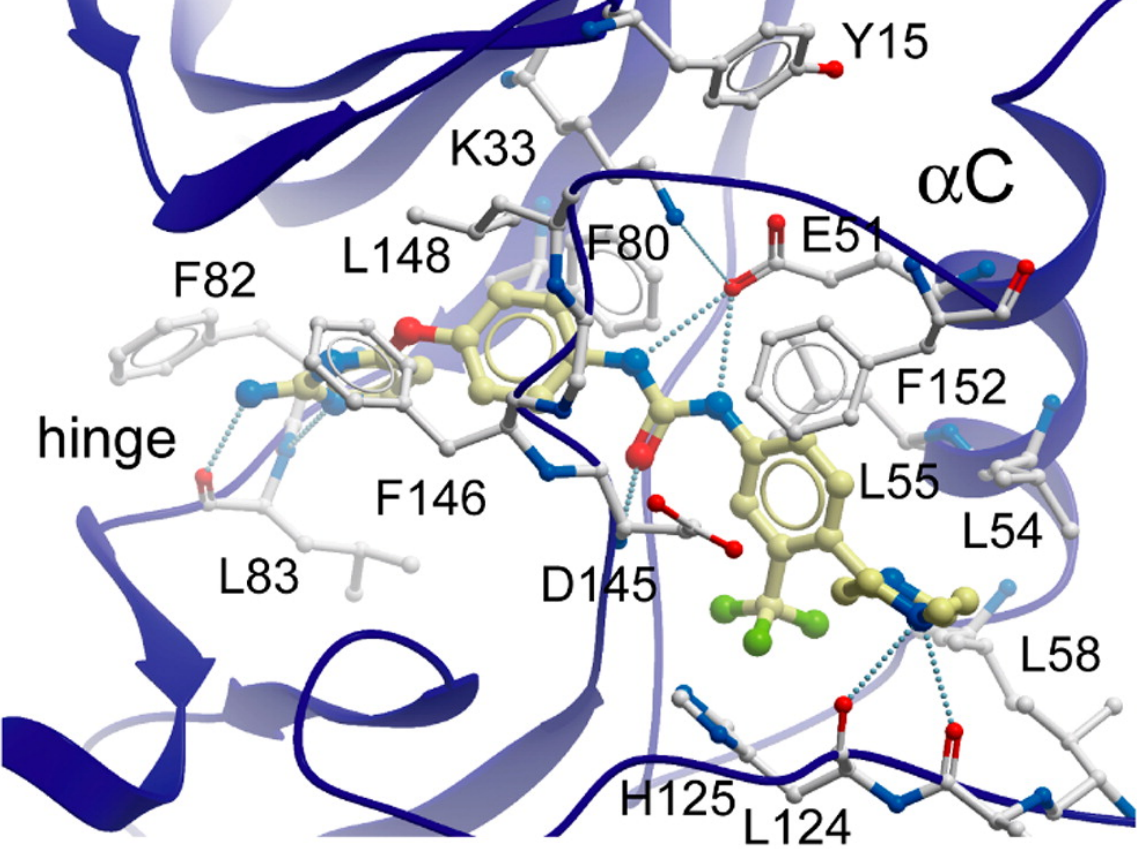

Supplement: fig_S4_ioaa107 [file fig_s4_ioaa107.pdf]

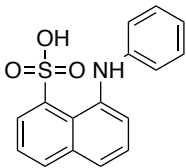

**ANS**

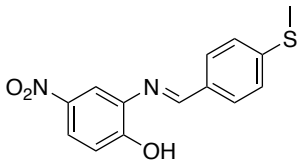

**B2**

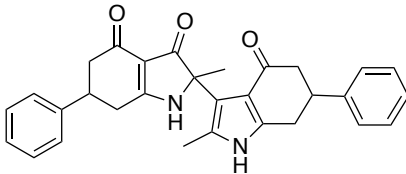

**DPIT**

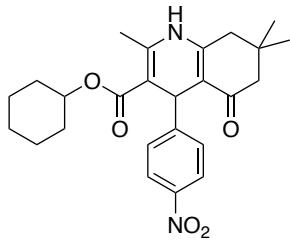**FLI-06**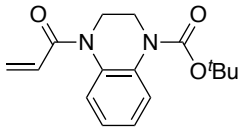

### Compound 1

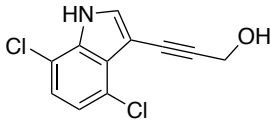

### Compound 2

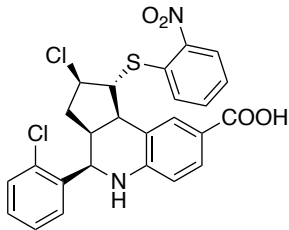

### Compound 3f

Supplement: fig_S5_ioaa107 [file fig_s5_ioaa107.pdf]

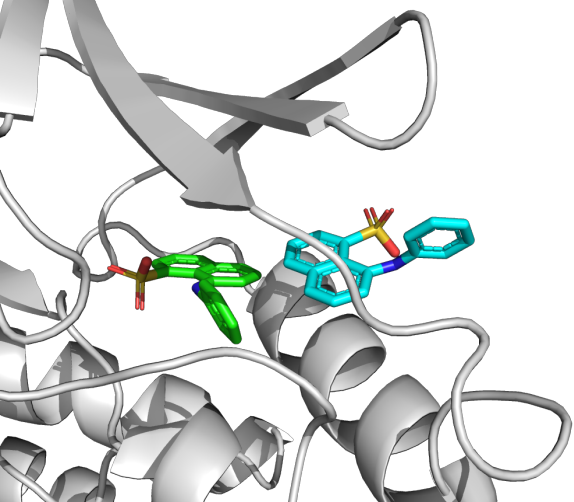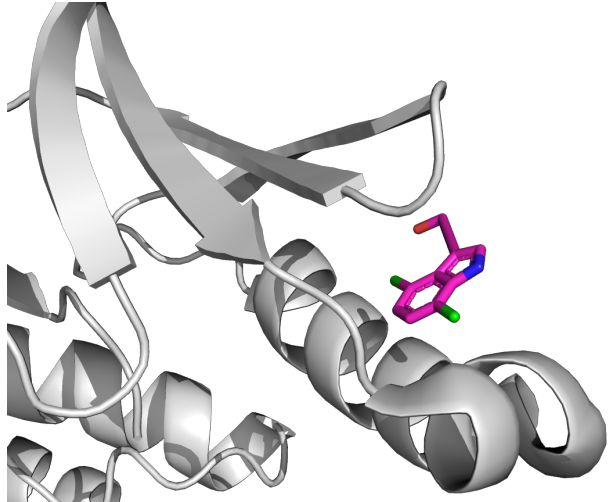

Supplement: fig_S6_ioaa107 [file fig_s6_ioaa107.pdf]
